# Blocking screw augmentation in intramedullary nailing for displaced surgical neck fractures of the proximal humerus

**DOI:** 10.1016/j.xrrt.2021.09.011

**Published:** 2021-11-12

**Authors:** Noboru Matsumura, Ryogo Furuhata, Takumi Nakamura, Hiroo Kimura, Taku Suzuki, Takuji Iwamoto

**Affiliations:** Department of Orthopedic Surgery, Keio University School of Medicine, Tokyo, Japan

**Keywords:** Proximal humeral fracture, Surgical neck fracture, Intramedullary nail, Osteosynthesis, Blocking screw, Poller screw

## Abstract

A displaced surgical neck fracture can be a good indication for antegrade intramedullary nailing. However, nail insertion may result in malreduction and translational displacement of the humeral head fragment because of muscle traction and size discrepancies between the diameters of the medullary canal and the intramedullary nail. We used blocking screw augmentation in 20 fractures with residual medial displacement of the distal fragment after nail insertion to anatomically reduce displacement of the fracture and to maintain the reduced position before bone union. A blocking screw was placed percutaneously at the lateral side of the canal. Next, a straight intramedullary nail was reinserted medial to the blocking screw. Finally, the nail was locked both proximally and distally. All cases showed bone union without fixation failure at the time of the final follow-up. Blocking screw augmentation with intramedullary nailing is feasible for the treatment of humeral surgical neck fractures and is thought to be helpful for fracture reduction during surgery and stable fixation after surgery.

Proximal humeral fractures are common in elderly persons with osteoporosis.^4^^,^^21^ Although most of them can be treated nonoperatively, displaced and unstable fractures often require surgical intervention.^1^ Locking plate fixation is the most commonly used fixation option and can provide stability for fragmented fractures.^12^ On the other hand, antegrade intramedullary nailing is less invasive to the periosteum and blood vessels, and it can be an alternative treatment option.^5^^,^^24^ In particular, a displaced 2-part surgical neck fracture in accordance with the Neer classification,^19^ in which the greater and lesser tuberosities are not fragmented or displaced, can be a good indication for intramedullary nailing.^3^^,^^12^ Intramedullary nailing is reported to be equivalent or superior to locking plate fixation in reducing complication rates for 2-part surgical neck fractures.^22^^,^^24^^,^^27^

Although newer-generation straight nails were reported to reduce the incidence of complications and reoperations compared with early bent designs,^3^^,^^18^ straight nailing for 2-part surgical neck fractures was reported to still be associated with an unacceptable rate of secondary displacement.^23^ In completely displaced surgical neck fractures, the distal fragment of the humeral shaft is displaced medially because of the muscle pull of traction by the pectoralis major, latissimus dorsi, and teres major muscles, whereas the proximal humerus is usually maintained at the normal position and with normal tilt, due to the balanced forces of the rotator cuff.^3^ However, the humeral medullary canal at the level of the fracture site is relatively wide compared with the diameter of the nail. Thus, there are size discrepancies between the diameters of the medullary canal and the nail. Even when the nail’s entry point is created at the top of the humeral head, nail insertion may result in malreduction and translational displacement of the humeral head fragment lateral relative to the shaft.^17^ Malreduction and insufficient medial cortical support can be risk factors for fixation failure and varus collapse.^11^^,^^13^ Anatomical reduction and stable fixation of the fragments can lead to good clinical results in cases with proximal humeral fractures.

We have used intramedullary nails for displaced surgical neck fractures of the proximal humerus. When medial translation of the humeral shaft remained and the fracture was not sufficiently reduced after nail insertion, blocking screw augmentation was added to anatomically reduce displacement of the fracture and to maintain the reduced position before bone union.

## Surgical technique

All surgeries were performed under general anesthesia with an interscalene block (Video 1). Patients were placed in the beach chair position. Through a 5-cm incision on the anterolateral side of the shoulder, a deltoid-splitting approach between the anterior and middle portions of the deltoid was used. After the deltoid muscle was bluntly split, the supraspinatus muscle was divided through its muscle fibers. In cases with rotator cuff tears, the nail was inserted through the rotator cuff tear site. The fracture was reduced under fluoroscopic control. When angulated deformity remained, a Kirschner wire (1.8 mm in diameter) was inserted into the humeral head to rotate the proximal fragment as a joystick.^25^ To obtain head anchoring of the fixation at the top of the humeral head^7^^,^^17^ and to avoid iatrogenic rotator cuff tears,^3^^,^^6^ a straight nail was selected.^18^ The entry hole was created at the top of the humeral head by pulling the multiple traction sutures placed in the rotator cuff, and an antegrade interlocking nail was inserted under fluoroscopic control at the adducted shoulder position. However, if medial translation of the humeral shaft remained, and the fracture was not sufficiently reduced, blocking screw augmentation was added.

**Figure 1:**
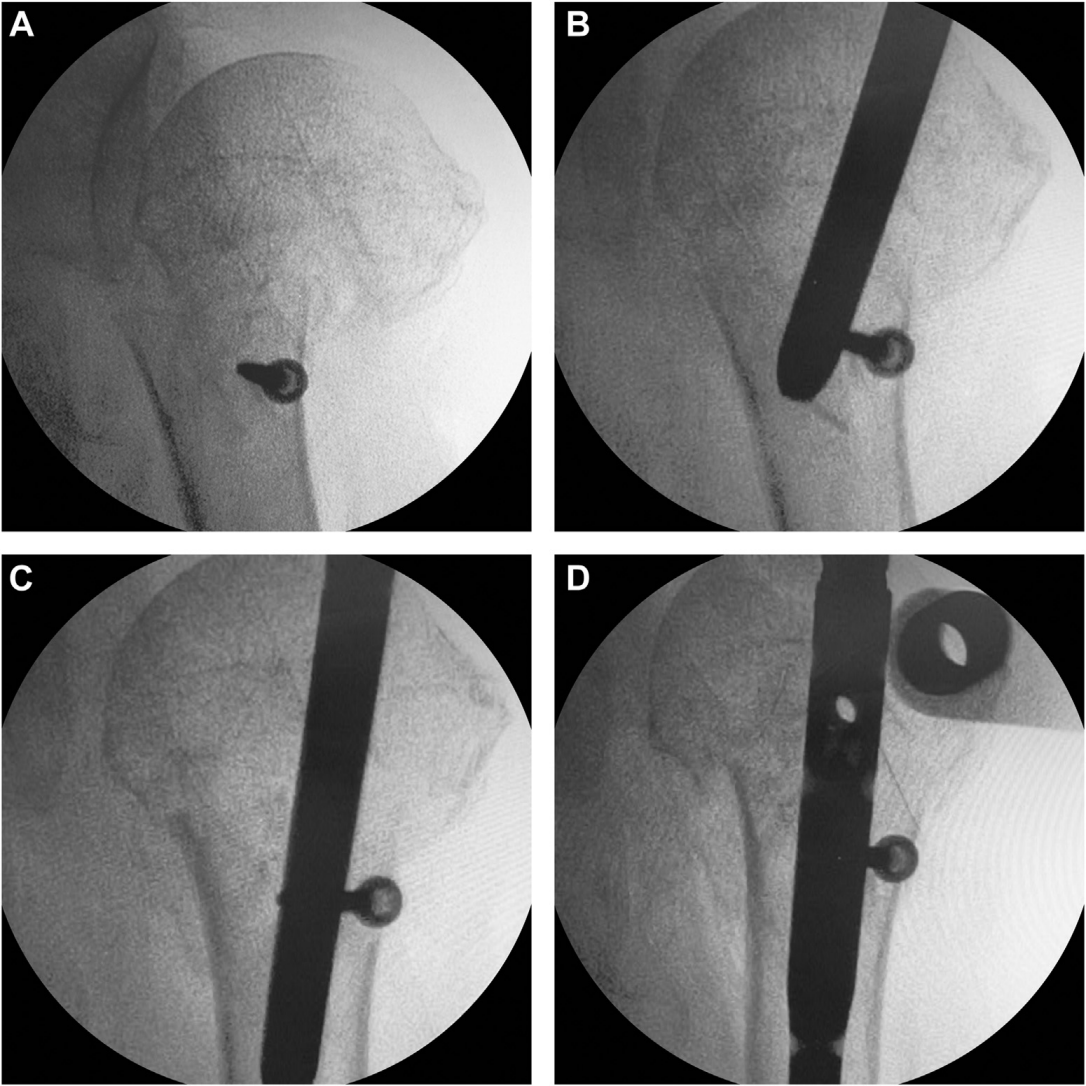
Blocking screw augmentation for intramedullary nailing. (**A**) The blocking screw placed at the lateral one-third of the distal fragment. (**B**) Antegrade nail insertion in the medullary canal medial to the blocking screw. (**C**) Blocking screw augmentation helping fracture reduction by lateral translation of the humeral shaft. (**D**) Successful reduction of the surgical neck fracture by nail insertion to an adequate depth.

The nail was removed, and the blocking screw was placed before nail reinsertion. The blocking screws were always inserted at the distal fragment because the proximal fragment is more fragile and comminuted compared with the distal humeral shaft. Through a small 1-cm incision on the anterior shoulder, a 3.5-mm-diameter cortical screw was percutaneously inserted as a blocking screw into the distal fragment at the level of more than 1 cm away from the fracture site after bicortical drilling of the bicortical bone. To control fracture reduction in the medio-lateral direction, the screw was placed at the lateral one-third of the shaft, and care was taken to keep the arms at neutral rotation during screw insertion. Then, an intramedullary nail was reinserted in the medullary canal medial to the blocking screw. As the actual width of the intramedullary canal for the nail was shortened, the screw helped shift the humeral shaft laterally (Fig. 1). After nail insertion, multiple proximal interlocking screws were fixed, followed by 2 distal interlocking screws in neutral shoulder rotation. After confirmation of fracture fixation under fluoroscopy, the split supraspinatus and deltoid muscles were repaired, followed by skin wound closure. For cases with repairable rotator cuff tears, the ruptured tendons were repaired using transosseous technique; for cases of irreparable tears, no repair was performed.

Postoperative rehabilitation was performed irrespective of the co-existence of a rotator cuff tear. The arm was put in a sling after osteosynthesis. Stooping exercise, pendulum exercise, and passive shoulder range of motion exercise were started one week after surgery. Then, active shoulder motion was allowed four weeks after surgery. Sports activity, heavy work, and weight-bearing were allowed four months after surgery.

## Results

During the period between October 2011 and April 2019, 50 patients with displaced surgical neck fractures of the proximal humerus were treated with straight interlocking nailing. However, as nail insertion could not successfully reduce medial translation of the humeral shaft in 23 cases, blocking screw augmentation was added. In one case of an 82-year-old woman, the blocking screw was displaced during nail insertion, and the fracture was fixed by intramedullary nailing without blocking screw augmentation. Two cases were lost to follow-up despite successful fixation of the fracture. Thus, a total of 20 patients (6 men and 14 women) with a minimum postoperative follow-up period of 12 months were retrospectively evaluated (mean, 24.7 ± 12.5 months; range, 12-50 months). Three patients had a smoking history. One patient was a present smoker, and 2 patients were former smokers who had quit smoking more than 10 years before the fracture. The mean age of the patients at the time of surgical fixation was 70.5 ± 13.9 years (range, 39-89 years). Surgical intervention was performed by a single surgeon on an average of 9.5 ± 4.8 days (range, 3-19 days) after the injuries. A solid AESCULAP Targon PH^+^ nail (B. Braun, Melsungen, Germany) was used in the 7 earlier cases, whereas a cannulated TURIUS humeral nail (Medical Engineering System, Inc., Tokyo, Japan) was used in the 13 later cases. Both nails were 150 mm in length, and the diameter of the nails was 10 mm in the proximal part and 8 mm in the distal part. The mean number of proximal interlocking screws was 4.1 ± 0.7 (range, 3-5), and the mean operative time was 67.9 ± 17.0 minutes (range, 48-106 minutes). During surgery, 5 cases were noted to have rotator cuff tears, which were thought to have existed before the fracture (mean age, 79.0 ± 9.4 years; range, 67-89 years). The ruptured tendons were repaired in 4 cases that were repairable, and no repair was performed in 1 case with an irreparable massive tear.

**Figure 2:**
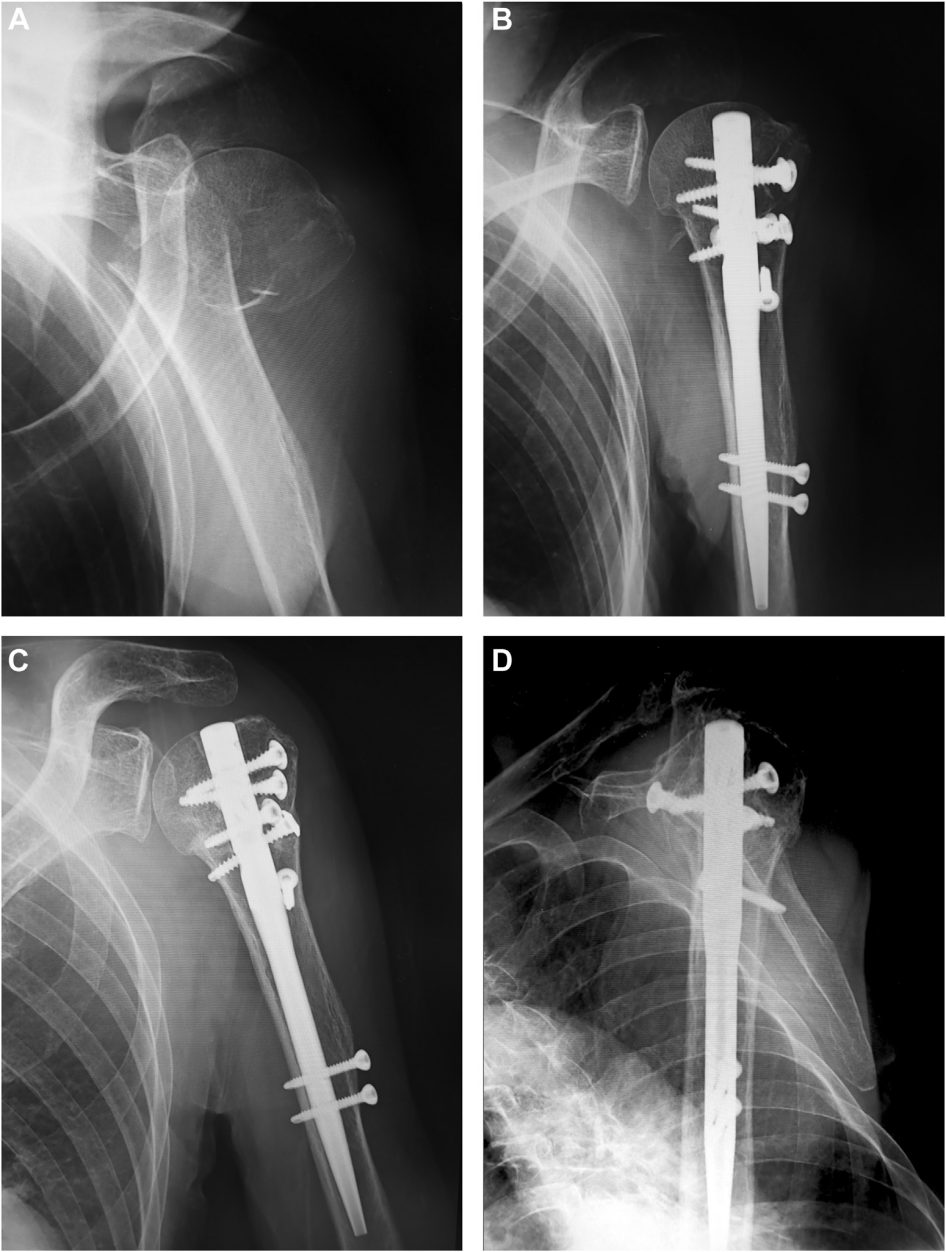
*Left* humeral surgical neck fracture of an 87-year-old woman. (**A**) Anteroposterior radiograph at the time of injury. The distal fragment shows medial translation of 65.4%, and the preoperative neck-shaft angle is 168°. (**B**) Anteroposterior radiograph 2 weeks after osteosynthesis. Intramedullary nailing with blocking screw augmentation reduces medial translation, and the neck-shaft angle is 142°. (**C**) Anteroposterior radiograph at the final follow-up 12 months after osteosynthesis. Bone union is achieved without any correction loss. (**D**) Scapular-Y radiograph at the final follow-up. Posterior angulated deformity does not remain.

**Figure 3:**
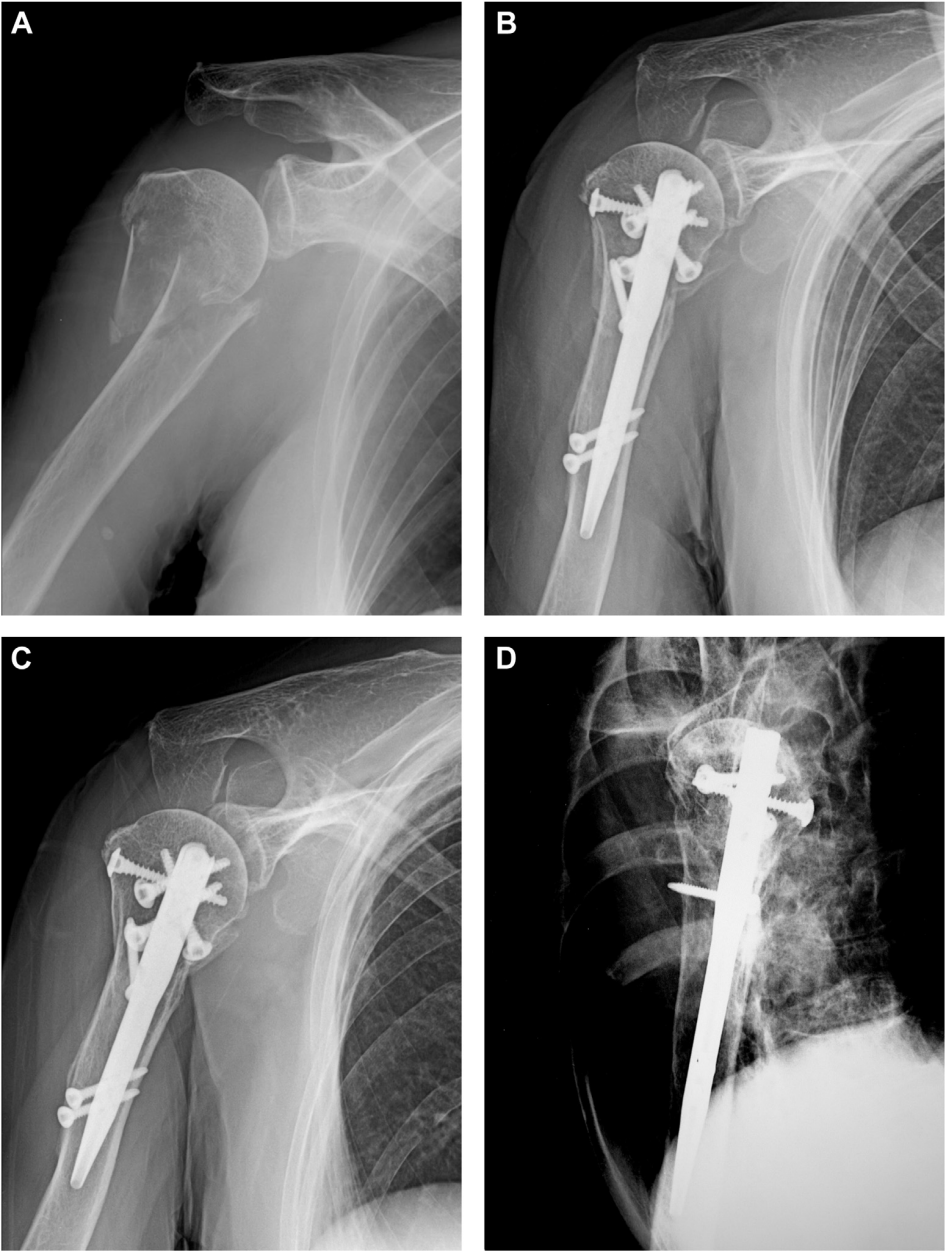
*Right* humeral surgical neck fracture of an 88-year-old woman. (**A**) Anteroposterior radiograph at the time of injury showing medial displacement of the humeral shaft and a wedge fragment in the metaphysis. (**B**) Anteroposterior radiograph 2 weeks after osteosynthesis. Intramedullary nailing with blocking screw augmentation reduces medial translation, and the neck-shaft angle is 140°. (**C**) Anteroposterior radiograph at the final follow-up 15 months after osteosynthesis. Bone union is achieved without any correction loss. (**D**) Scapular-Y radiograph at the final follow-up. Posterior angulated deformity does not remain.

All cases showed bone union without fixation failure at the time of the final follow-up (Figs. 2 and 3). Although back out of the most distal interlocking screw was found on the radiographs in the case of an 89-year-old woman at the final follow-up, the screw remained in the bone and did not need to be removed. No patient had infection, ipsilateral nerve palsy, evidence of nonunion or avascular necrosis of the humeral head, or other complications. The mean active shoulder range of motion at the time of the final follow-up was 131 ± 15° (range, 90-150°) in elevation, 39 ± 17° (range, 10-80°) in external rotation at the side, and 9th ± 1 thoracic vertebral level (range, 8th thoracic vertebral level-1st lumbar vertebral level) in internal rotation behind the back. The mean American Shoulder and Elbow Surgeons Shoulder score (ASES) at the final follow-up was 84.6 ± 8.4 points (range, 67-100 points). The mean ASES score was 79.2 ± 5.9 points (range 73-85 points) in cases with repairable rotator cuff tears and 87 points in the case with an irreparable tear.

The medial translation ratio and neck-shaft angle on the anteroposterior view of plain radiographs were compared before surgery, just after surgery, and at the final follow-up using Friedman tests. Posterior angulation was evaluated as the angle between the axes of the humeral shaft and the proximal humerus on the scapular-Y radiographs. With significant effects, post hoc Wilcoxon signed-rank tests with Bonferroni correction were performed to identify significant differences. The mean width of the medullary canal was 28.7 ± 3.3 mm (range, 25.0-36.4 mm) at the level of the fracture site and 15.3 ± 1.7 mm (range, 12.2-18.9 mm) at the level of the deltoid tuberosity. The average medial translation was 16.5 ± 4.6 mm (range, 10.1-28.2 mm), and its ratio was 57.5% ± 15.9% (range, 38.1-105.3%). The preoperative neck-shaft angle was widely distributed, from 114° to 168°, with a mean value of 149 ± 14°. The mean posterior angulation at the time of injury was 15 ± 13° (range, −8 to 41°) on scapular-Y radiographs. The medial translation ratio was significantly decreased by an average of 0.1% ± 4.9% (range, −16.6 to 6.1%) after osteosynthesis (*P* < .001). The mean neck-shaft angle and posterior angulation were also significantly decreased to 138 ± 4° (range, 133-144°) (*P* = .010) and 2 ± 3° (range, −8 to 7°) (*P* < .001), respectively. At the final follow-up, all 20 fractures united without obvious postoperative correction loss, with a mean medial translation ratio of −0.5% ± 5.7% (range, −13.0 to 9.6%) (*P* = 1.000), mean neck-shaft angle of 137 ± 5° (range, 127-145°) (*P* = 1.000), and mean posterior angulation of 1 ± 3° (range, −9 to 6°) (*P* = 1.000).

## Discussion

In this study, a transmedullary blocking screw, also known as a Poller screw,^14^^,^^15^ was used with an intramedullary nail to aid in reduction of displaced 2-part proximal humeral fractures.^26^ The most frequent indication for the use of this screw is in oblique long bone fractures at the metaphyseal-diaphyseal junction.^10^ Unlike for diaphyseal fractures, nailing of metaphyseal fractures with a short proximal or distal fragment is associated with an increase in malalignment because of displacing muscular forces and residual instability.^8^ As there is a large difference between the size of the implant and the metaphyseal diameter with no nail-cortex contact, the nail may translate in the medullary canal.^15^ The screw is thought to work by narrowing the medullary canal in the metaphysis to provide a tight mechanical fit for the intramedullary nail.^26^ Stedtfeld et al^26^ reported the use of this technique for a case with a spiral humeral shaft fracture. However, no report has ever focused on blocking screw augmentation for the treatment of proximal humeral fractures, which are the third most common fractures and are increasing in frequency.^9^^,^^21^

The average width of the intramedullary canal was 28.7 mm at the level of the fracture site and 15.3 mm at the level of the deltoid tuberosity, whereas the diameter of the intramedullary nails used in the present cases was 10 mm in the proximal part and 8 mm in the distal part. Thus, the intramedullary canal was almost 3-times wider at the level of the surgical neck and 2-times wider at the midshaft of the humerus than the intramedullary nail, representing a large size discrepancy between the diameters of the medullary canal and the nail. In a typical displaced surgical neck fracture, in which the proximal and distal fragments are completely displaced, the proximal humerus is sustained at its original position and the humeral shaft is pulled medially by the traction of the muscles originating from the thorax or scapula and inserting to the humeral shaft. In the present cases, blocking screw augmentation on the lateral aspect of the humeral shaft kept the nail placement medially in the humerus and translated the distal fragment laterally relative to the proximal fragment. The blocking screw was bi-cortically inserted on the lateral aspect of the distal fragment, and the screw itself appeared to be stabilized strongly between the intramedullary cortical wall and the nail after nail insertion. In 23 displaced surgical neck fractures, the intramedullary blocking screw successfully supported the nails in all but one fracture. The present cases showed that this augmentation technique is feasible and useful for anatomical reduction in intramedullary nailing for humeral surgical neck fractures.

Even when good fracture reduction can be obtained during osteosynthesis, postoperative correction loss can occur.^20^ Proximal humeral fractures predominantly affect elderly women,^21^ and bone quality is not always sufficient for stable fixation.^11^^,^^13^ Temporary wire insertion instead of a blocking screw, which could be easier than screw placement, is also used for precise positioning of nails.^2^ However, blocking screw augmentation can work not only on fracture reduction during surgery but also on increasing stability of the bone-implant construct after surgery.^16^ Use of transmedullary blocking screws with an intramedullary nail can achieve three-point fixation, which consists of the entrance point of the nail, blocking screw, and the cortical wall through the isthmus.^26^ Furthermore, head anchoring has been reported to be important for stable nail fixation in cases with proximal humeral fractures,^6^^,^^7^ and blocking screw augmentation can sustain the nail’s proximal end in the zone of dense subchondral bone in the proximal humerus.^17^ In the present cases, all fractures united successfully without correction loss, leading to satisfactory clinical outcomes despite the old age of the patients.

One limitation of the present study was the lack of a control group. As good results were reported for straight intramedullary nails in the treatment of humeral surgical neck fractures,^3^^,^^18^ the necessity of blocking screw augmentation was not demonstrated. Second, blocking screw augmentation was added in cases with residual medial displacement of the distal fragment even after nail insertion. This could create selection bias. Furthermore, we tried to anatomically reduce the proximal humeral fractures, but whether better anatomical reduction can yield better clinical results in intramedullary nailing remains unknown. Further study will be needed to clarify the importance and effectiveness of blocking screw augmentation with intramedullary nailing for the treatment of displaced surgical neck fractures of the proximal humerus.

## Conclusion

Blocking screw augmentation in intramedullary nailing is feasible for the treatment of humeral surgical neck fractures and is thought to be helpful for both fracture reduction during surgery and stable fixation after surgery.

## Disclaimers

Funding: No funding was disclosed by the authors.

Conflicts of interest: The authors, their immediate families, and any research foundation with which they are affiliated have not received any financial payments or other benefits from any commercial entity related to the subject of this article.

## Supplementary data

Video 1 Intramedullary nailing with blocking screw augmentation technique for displaced surgical neck fractures of the proximal humerus.
